# Improving symptom assessment and management in the community through capacity building of primary palliative care: A study protocol of exploratory research

**DOI:** 10.12688/f1000research.111644.1

**Published:** 2022-07-01

**Authors:** Malathi G Nayak, Radhika R Pai, Baby S Nayak, Sudhakara Upadya P, Naveen Salins

**Affiliations:** 1 Department of Community Health Nursing, Manipal College of Nursing, Manipal Academy of Higher Education, Udupi, Karnataka, 576104, India; 2 Department of Fundamentals of Nursing, Manipal College of Nursing, Manipal Academy of Higher Education, Udupi, Karnataka, 576104, India; 3 Department of Child Health Nursing, Manipal College of Nursing, Manipal Academy of Higher Education, Udupi, Karnataka, 576104, India; 4 Manipal School of Information Sciences, School of Information Science, Manipal Academy of Higher Education, Udupi, Karnataka, 576104, India; 5 Department of Palliative Medicine and Supportive Care, Kasturba Medical College, Manipal Academy of Higher Education, Udupi, Karnataka, 576104, India

**Keywords:** ASHA (Accredited Social Health Activist), Capacity building, Field Health Assistants, Junior Health worker, Palliative Care, Symptoms, Primary Health Care workers, Nurses

## Abstract

**Aim:** To determine the effectiveness of capacity building program on palliative care (PC) in enhancing the capacity of the primary health care workers in need assessment and symptom management of cancer patients.

**Background:** In India, less than one percent of people living with cancer have access to palliative care since most are from low- and middle-income groups. Accredited social health activist (ASHA) and primary health care workers are grassroots workers who are the first contact with family members and are seldom aware of PC in India. It is essential to train them to give practical and efficient care to needy people.

**Design:** A quasi-experimental design with follow-up will be conducted using an evaluative approach.

**Methods:** The study population consists of 1440 Primary Health Care Workers (staff nurses, ANMs, and ASHA workers) of three taluks of Udupi District, Karnataka State, India. Training on PC will be provided for ASHA workers for one day and ANM/Staff nurses for three days. After their training, they are expected to demonstrate the gain in knowledge & skill in providing PC for cancer patients by identifying and implementing PC services using a mobile app at the primary healthcare level.

**Discussion:** Palliative home care can give comfort and reduce patients’ financial burden, and this training may help to improve the quality of life of needy patients.

**Impact:** If this palliative care training program succeeds, it can be integrated into the healthcare continuum, making it an essential component of primary healthcare delivery to achieve universal health coverage. Moreover, home-based PC supports patients who want to die at home even though it reduces hospital stay costs.

**Trial registration:** CTRI/2020/04/024792.

## Introduction

The GLOBOCAN 2020 estimated 19.3 million new cases of cancer and 10 million bereavements due to cancer in 2020 ( Sung *et al.*, 2021). Both private and public primary care facilities and public secondary facilities are inadequately prepared to address the burden of non-communicable diseases (NCDs) in India ( Krishnan *et al.*, 2021). Inequitable access to palliative care is one of the greatest disparities in global health care and lack of access to palliative care in low- and middle-income countries (LMICs) has led to a huge burden of preventable suffering ( Knaul, Bhadelia, Rodriguez, Arreola-Ornelas, & Zimmermann, 2018). Although there are several initiatives towards palliative care development in India, palliative care access is significantly limited to a small population ( Rajagopal, 2015). Improving palliative care education and awareness of healthcare professionals in India might be an effective strategy towards facilitating palliative care access ( Glass *et al.*, 2020).

According to the oncology-based palliative care development project designed for the north-eastern region of India, developing oncology-based palliative care in India could demonstrate the implementation of a local version of the WHO model which emphasizes highlights the strengths of integrating palliative care within the cancer care programs right from its inception. It highlights the supportable public health care services present in comparison with the funded initiatives. This WHO model emphasizes the usefulness of this for LMIC countries with similar health and socio-cultural contexts ( Vallath *et al.*, 2021). In this regard, India requires the development of clinical guidelines according to the local needs for palliative home care ensured quality and uniform delivery of clinical services ( Jeba *et al.*, 2020).

Helping native populations to institute community-led palliative care programs is the best way to address the lack of accessibility and sustainability of appropriate palliative care services at the end of life to most people in the world which is a complex task. This requires great rigor in both stages of conceptualization and implementation ( Kumar, 2020).

Capacity building of community health care workers in Brazil yielded in the successful conduct of the home visits and screening for risk factors in the elderly population ( Neto *et al.*, 2021). Nurses play a vital role in the care of patients especially caring for those who are dying, hence it is imperative for them to know the best principles of palliative care ( Sadhu, Salins, & Kamath, 2010). The role of the family in the care of chronically ill patients is significant and they should be socially supported and empowered to cope with the situation ( Philip, Philip, Tripathy, Manima, & Venables, 2018).

Availability of the digital health device for the reporting of the symptoms in patients with cancer to attend palliative care needs results in the reduction of the number of emergency department visits and thereby reduced health care costs ( Bhargava *et al.*, 2021). There is promising evidence to use mobile health apps to provide expanded and enhanced health care services to individuals and communities, taking advantage of the growing usage of mobile phones among diverse populations ( Källander *et al.*, 2013).

An India-specific document and a supportive quality development program suggested the need for additional training of palliative care providers towards the implementation of palliative care ( Salins, Johnson, & Macaden, 2017). Despite having a robust palliative care policy in India, a study carried out through direct fieldwork and interviews with the health care workers reported a lack of public health approach to end-of-life care. This study further suggested the need for increased participation of the local and political readiness to implement palliative care delivery effectively ( Jayalakshmi & Suhita, 2017).

National Health Systems Resource Centre (NHSRC) in India at present is associated with the preparation and training focussing on the Health care workers including ASHA, ANM to Medical Officers at Health and Wellness Centers across India ( Centre, 2021b). To build a liaison between the community and public health system in India, under National Rural Health Mission there are Accredited Social Health Activists (ASHA) selected from the village who is planned to gather individuals’ understanding of health rights and empower them to get to their privileges ( Centre, 2021a).

## Background

The Global Atlas reveals that over 56.8 million people need Palliative Care (PC) worldwide (Organization). Adults over 50 years’ account for 67.1%, with children accounting for 7.1%. Among the adults, the largest single disease group needing PC is cancer (28.2%). Among children, cancer accounts for 4.1% of PC needs. Across resource settings, 76% of adults and >97% children (0-19 years) are from low and middle-income countries (LMIC). The regional distribution shows that 17.1% of adults and 19.5 of children needing PC are from the South East Asian Region (SEAR). SHS amenable to PC interventions is expected to increase by 87% by 2060 ( Connor & Sepulveda Bermedo, 2018).

One of the key objectives of the National Programme for the prevention and control of cancer is establishing and developing a capacity of health care workers for planning health care activities including home care and palliative care ( DIRECTORATE GENERAL OF HEALTH SERVICES & Welfare). One of the aims of the national palliative care program is to improve the capacity among health care workers to provide palliative care service delivery within government health programs ( DIRECTORATE GENERAL OF HEALTH SERVICES & Welfare, 2012). Trained ASHA workers in their respective localities were able to identify people with psychiatric disorders and refer them to advanced care settings for further management ( Ibrahim *et al.*, 2021). Palliative care intervention that influences the inclusion of community health workers could serve as a feasible model for expanding the reach of palliative care to rural underserved patients ( Qanungo *et al.*, 2021). Rethinking of normal hospice services is recommended as they can participate in community capacity building in integrating palliative care ( Paul, Cree, & Murray, 2019).

The process of translating a palliative care plan into action requires strong leadership, competent management, political support, and integration across all levels of care ( Kar, Subitha, & Iswarya, 2015). CanSupport a multidisciplinary team approach reported that a home-based care model could be more approachable to the patients in India especially during end-of-life care or among patients with a chronic life-limiting illness. It is even reported in Can Support that the successful implementation could reduce the number of hospital visits, and finally reduce the total cost of the hospital by providing the best possible care by the trained health care people ( Yeager *et al.*, 2016).

Developing policies concerning inculcating family-centered palliative care and end-of-life care within the nursing curriculum could help in promoting the nurse-led community models to address the palliative and end-of-life service care in India ( Ramasamy Venkatasalu *et al.*, 2018).

There is a disparity between the number of patients who could be benefiting from palliative care and the number of palliative care specialists. It is a vital aspect to improve basic palliative care delivery skills, identification of patients in need, and appropriate referral to palliative care specialists to effectively integrate the same into primary health care ( Gorman, 2016).

Organizing home-based palliative care services as a quality improvement project resulted in a consistent increase in the frequency of home visits, better documentation, coordination, and accountability. Interdisciplinary team coordination helped to develop trust and a better understanding of collaborative research work ( Viswanath *et al.*, 2021).

The implementation of the Neighbourhood Network in Palliative Care (NNPC) in the Indian state of Kerala included the trained volunteers who were encouraged to form groups of 10-15 community volunteers, to identify the problems of the chronically ill people in their area and organize appropriate interventions. This NNPC service was reported as a cost-effective option for most developing countries to develop much-needed sustainable services for chronically ill and dying patients. In 2010, this network saw over 2500 patients per week and attained coverage of over 60% in many areas with NNPC groups that trained doctors and nurses support ( Sallnow, Kumar, & Numpeli, 2010). A home-based palliative care program was perceived to improve the lives of patients and their caregivers despite the challenges of maintaining a volunteer-led program ( Philip, Venables, Manima, Tripathy, & Philip, 2019).

Family carers suggested having a minimum number of carers involved in care, increasing or ensuring personal continuity, and maximizing the informational and organizational aspects of care could lead to a more positive experience ( Seamark *et al.*, 2014). There was some evidence of increased patient satisfaction with home-based end-of-life care ( Shepperd, Wee, & Straus, 2011).

A structured training program on symptom management kit for primary caregivers of cancer patients receiving home care was conducted at CMC, Vellore, India reported that, of the primary caregivers, 96.7% used a symptom management kit and received training resulted in a reduction of hospital visits for acute symptoms reduced by 80%; 90% were satisfied with the training received; 73% stated it was not a burden to treat the patient at home. This study further reported that the program and the kit were reported to be perceived favorably by caregivers from diverse backgrounds. Rural backgrounds and illiteracy were not barriers to acceptance of home-based palliative care ( Chellappan, Ezhilarasu, Gnanadurai, George, & Christopher, 2014).

The mobile phone was chosen because it helps CHWs achieve better results in their daily activities. The five main mobile health functions that support frontline health workers (FHWs) in providing adequate care to their communities are data collection and reporting, decision-making resources and training, emergency referrals, warnings and reminders, and supervision. also, mobile data collection increases data collection promptness, decreases error rates, improves data completeness ( Agarwal, Perry, Long, & Labrique, 2015).

Studies have shown that the training programs conducted for primary caregivers help provide palliative care. It also showed that most of them were satisfied with the training provided ( Chellappan *et al.*, 2014). A study was carried out to examine the effectiveness of a holistic capacity-building program for volunteers in community-based end-of-life care (EoLC). Following the screening, 88 of the 171 candidates completed core competency training, with 53 continuing to volunteer for the next six months. Their end-of-life care competence, self-care understanding, and death work competence improved significantly following training and remained stable at the 6-month follow-up ( Wang, Chan, & Lou, 2020).

A cross-sectional interventional study was conducted in Delhi on the effectiveness of the certificate course on essentials of palliative care program among 29 health care professionals. The study revealed that, in the pretest, about 24.1% of them had good knowledge and which was improved to 82.8% after the training. 62.1% had average knowledge, and only 13.8% had poor knowledge. Since completing the program, participants improved their communication skills, symptom management, negative news breaking, and pain assessment ( Bhatnagar & Patel, 2018). Studies also have reported that the scope and coverage of palliative care can be improved by providing training for medical officers and health care professionals and sensitizing the public through an awareness campaign ( Kar *et al.*, 2015).

This current project on improving the symptom assessment and management is intended to help to improve the knowledge and practice of primary health care workers on palliative care for need identification in the community.

## Protocol

### Research objectives

The objectives of the study are to:

• Assess the knowledge on palliative care pre and post-education among PHCWs by a survey study using a structured knowledge questionnaire.
• Assess the attitude on palliative care pre and post-education among PHCWs by a survey study using a structured attitude questionnaire.
• Assess the interpretive skill on palliative care pre and post-education among PHCWs by a survey study using a structured questionnaire on interpretive skill.
• Identify the challenges and barriers for delivery of palliative care at the community level through qualitative interviews of the focused groups using an interview topic guide.

### Study design

The impact of palliative care education on skills, attitude, and interpretive skills will be known through a Quasi-experimental design study having a pre-/post-intervention assessment of PHCWs (staff nurses, ANMs, and ASHA workers). The interventions will be the training of PHCWs on community palliative care and the implementation of a palliative care mobile app in the community. The outcome assessed is improvement in palliative care knowledge and enhancement of identification of palliative care needs in the community.

### Research setting and study population

The study will be conducted at 59 primary health centers and 316 sub-centers in the rural locations of a district in Southern India. The study participants will be eligible and consenting registered ASHA workers (1011) and Primary Health Care Workers who are working under Primary Health Centers and Sub Centers (staff nurses/ ANMs or Junior health workers/Assistants = 429).

### The rationale for selection of the participants

Cancer patients often experience financial constraints concerning cancer treatment in India ( Oswal *et al.*, 2021). As primary health care centers throughout the country deliver the essential medical assistance and the primary health care workers are the grass root level workers who work with the rural population closely, the national policy on palliative care emphasizes training the health care workers and providing accessible care to the cancer patients at the community level ( DIRECTORATE GENERAL OF HEALTH SERVICES & Welfare, 2012). World Health Organization (WHO) emphasizes all countries integrate palliative care at the primary health care level ( Organization, 2018).

**Figure 1.**
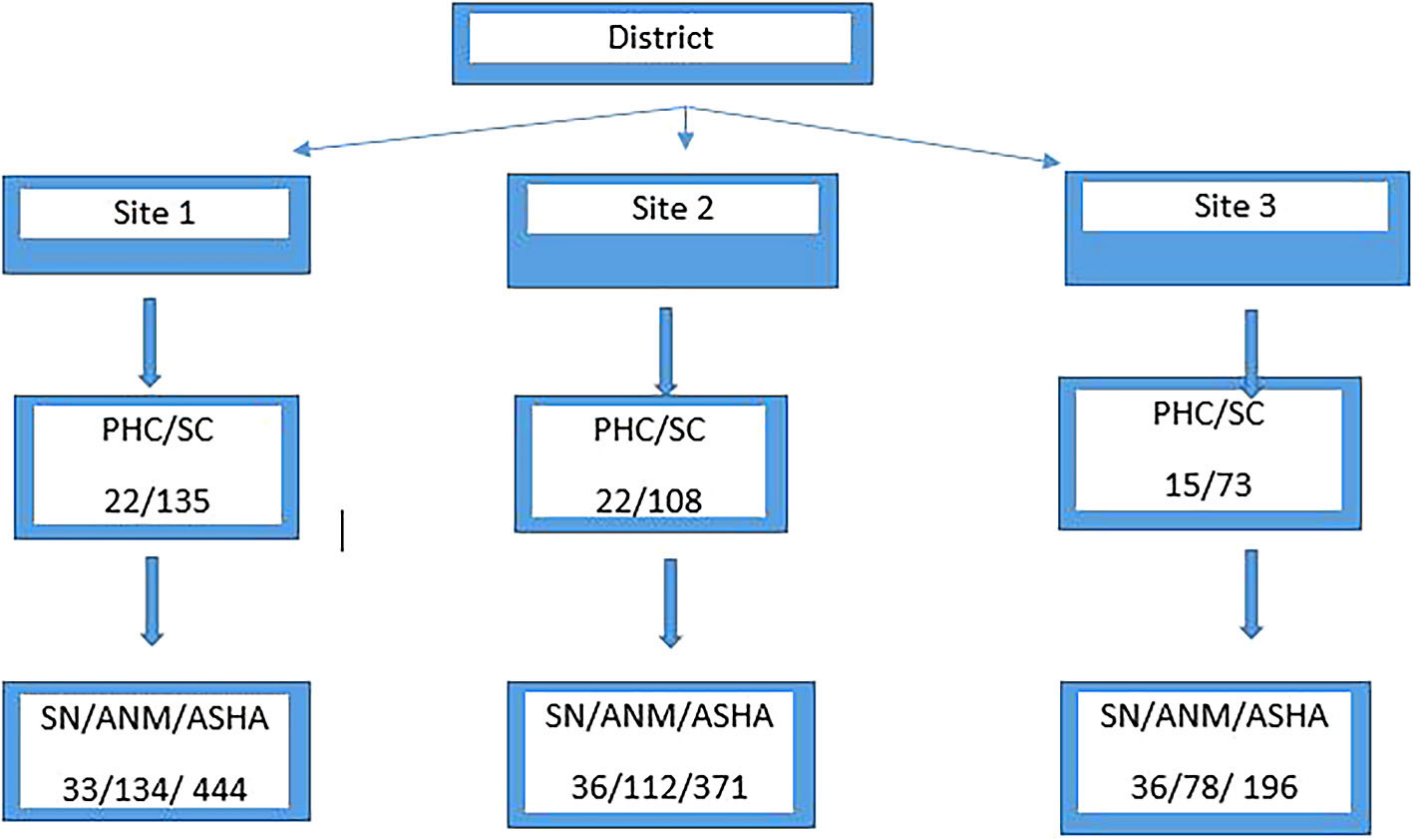
Number of SN/ANM/ASHAs working in PHC/SCs three taluk of selected District.

Thus the total population of PHCWs & ASHAs in the selected District is **1440** excluding vacancies ( Figure 1).

### Inclusion criteria

PHCWs working as Staff nurses, Auxiliary Nurse Midwives or Junior Health Assistants, and ASHA workers who are trained female community health activists who are selected from the same village to be an interface between the community and public health system working under PHC/SCs of Selected District will be the participant in this project. These workers who are willing to participate in the study will be trained to identify the symptoms requiring palliative care for children and adults with cancer and provide palliative care for the same.

### Intervention

**Training**

The palliative care module will be prepared and training will be provided to the PHCWs to sensitize them concerning the concept of palliative care. A series of education will be planned and it will be conducted through workshops.

### Development of palliative care module for PHCWs

The palliative care module for PHCWs will consist of:

• Introduction, definition, principles of palliative care
• The role of a nurse in palliative care
• Symptoms assessment, and management such as pain, dyspnoea, nausea and vomiting, diarrhea, constipation, wounds, edema, skin problems, anorexia, cachexia, fever.
• Practical sessions on colostomy care, nasogastric tube insertion and tube feeding, urinary catheterization and catheter care, oral care, tracheostomy care, wound care, prevention of bedsores, subcutaneous injection procedures, lymphedema care.

### Outline for mobile app

To enable the functioning and tracking, an Android-based Application will be developed for usage by the PHCWs and health service providers. The Android App would contain the following functional modules

i. Knowledge center that includes the information about palliative care needs, symptoms identification management. The content would contain Images, Videos, and links to online resources.
ii. Module to register, create and update the patient data
iii. Search and generate alerts for patient monitoring by Health workers
iv. Option to synchronize the data with the Health center for tracking purpose

**Figure 2.**
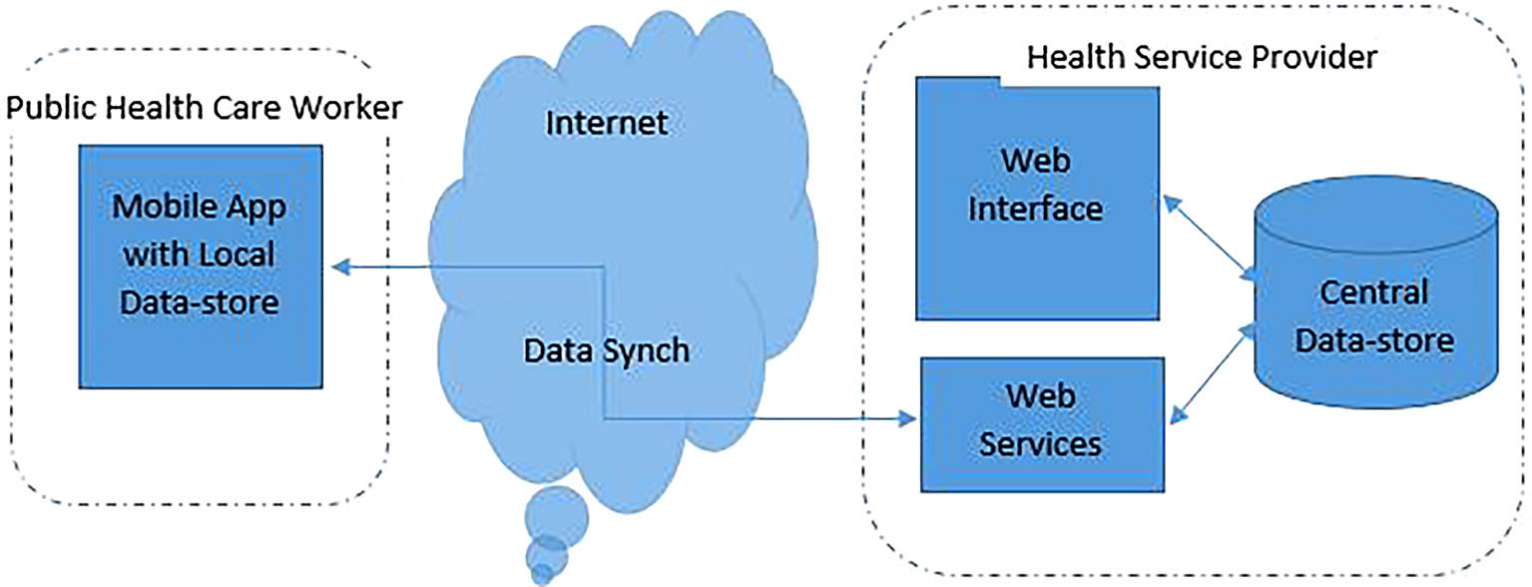
Application architecture.

The overall application structure will be as depicted in Figure 2.

The Public Health Care Worker (PHCW) will have an Android-based application on their mobile devices. The Android App features are as per the details outlined in Figure 2. The App can store the data locally and forward the details to the central data repository for monitoring and reporting purposes. Using the App on their mobile device, health worker refers to the training materials available in the App and also record the patient details of the locality they cover. The patient data collected by the health workers on the Android App will be transferred to the central data store by the health worker using the data synch option of the App.

On the Health Service Provider section, the application would contain a Web Interface, Web services, and the Central Data-store. The Web services section would help in registering the health worker details and configure the App on the mobile device of the health worker for the intended functioning ( Table 1). The web services will also enable data synchronization between the mobile device and central data store. Central Data-store acts as the repository of data collected from all the health workers and acts as the basis from which various findings from the study in the form of different reports can be generated. The Web interface provides the mechanism for the study managers at the Health Service Provider section to monitor the system and generate reports from the system.

**Table 1.** Outline for mobile app.

PHCWs details to be entered in mobile app	Identification of symptoms and management details
1. Login → User ID for ASHA/ANM/Staff Nurse (ID should begin from 01 for each category) → Password	1. Patient Id → Name of the patient → Age → Gender → Name of the city/village → Disease condition Symptom
2. Name of Taluk: Site 1, Site 2, and Site 3	2. No. of cases identified by the PHCW
3. Name of ASHA/ANM/Staff Nurse	3. No. of symptoms identified
4. Gender	4. No. of episodes occurred for each patient
5. Phone number	5. No. of follow up done
6. Name of health center: PHC/CHC/SC	6. Symptoms managed
	7. No. of cases referred
	8. No. of deaths → Case closed (In case of death)
	9. Feedback by patient and family members

### Expected outcome

The expected outcome of this study is to identify the impact of palliative care training in improving palliative care knowledge and their ability to assess palliative care needs in the community among PHCWs. To explore views of PHCWs on the facilitators and barriers for palliative care delivery in the community.

### Recruitment, consent, and data collection

**Figure 3.**
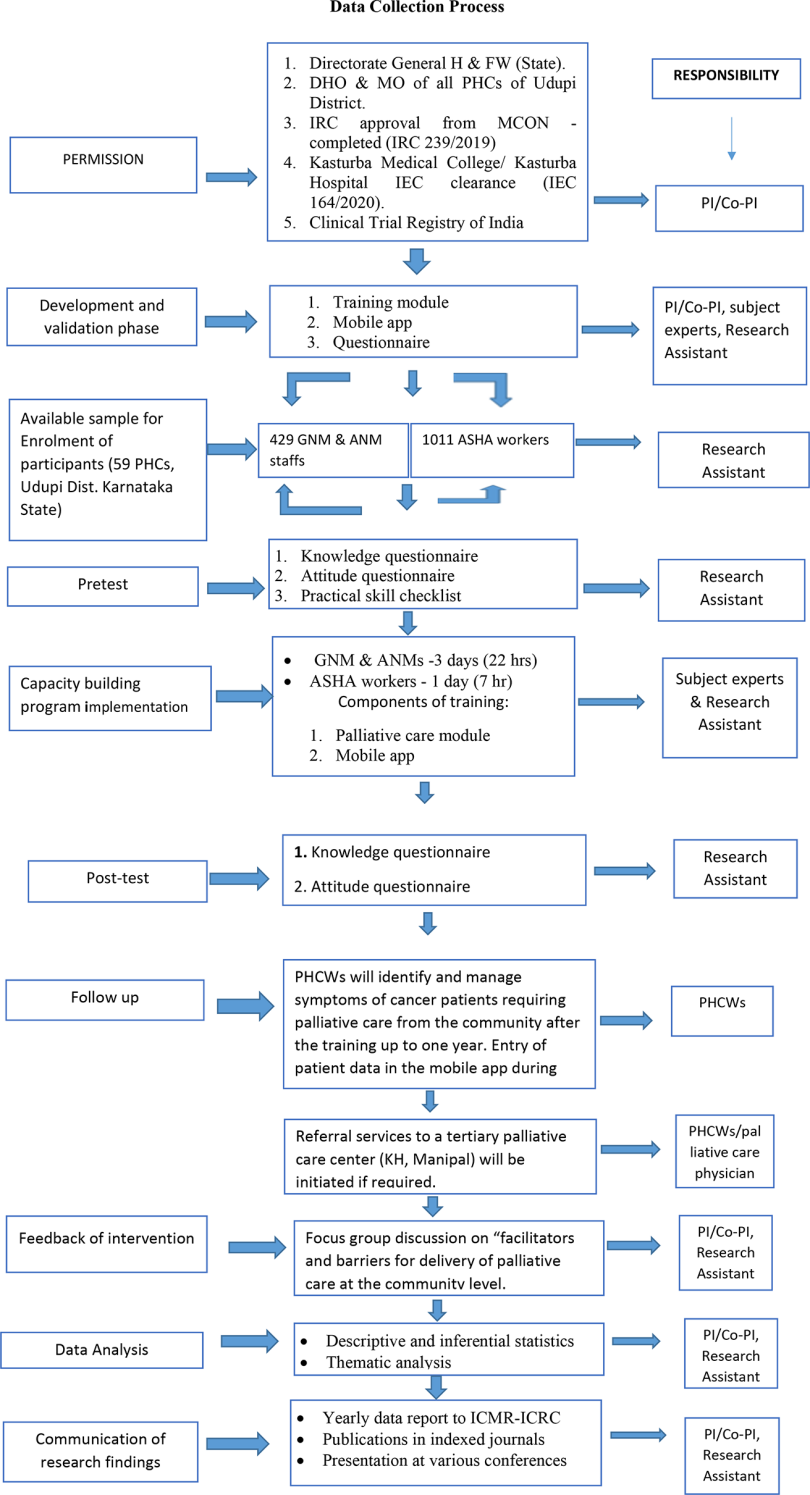
Schematic representation of data collection process.

All regulatory and administrative approvals will be sought. After explaining the procedure and objectives of the research project, written informed consent from all the participants will be obtained. The questionnaires are prepared in English and Kannada. The outcome of the project will be measured by using demographic proforma, knowledge questionnaire, attitude scale, skill checklist, and validated set of questions on focus group discussion ( Figure 3).

### Data collection instruments

The questionnaires to be used in this project include demographic proforma, structured knowledge questionnaire on palliative care, attitude scale, and checklist for skill assessment.

a) The demographic proforma will be used to assess the demographic characteristics of the PHCWs such as age, gender, marital status, religion, monthly income, designation, place of work, years of experience, and area of working.
b) A structured knowledge questionnaire will be used to assess the existing knowledge on palliative care among PHCWs. The questionnaire includes the areas like essential elements of palliative care, the role of the palliative care team, the role of the nurse in palliative care, nursing ethics, various symptom identification and management, communication skills, end of life care, grief & bereavement care by using an MCQs and dichotomous items and also open-ended items. The knowledge score for staff nurses, ANMs contains 30 MCQs. The scores are categorized as 26 and above (75%), 18 to 25 (50 to 74%), 17 and below (less than 50%), which indicates good knowledge, average knowledge, poor knowledge, respectively. The knowledge score for ASHA workers contains 22 items. The scores are categorized as 17 and above (75%), 11 to 16 (50-74%), ten and below (less than 50%), which indicates good knowledge, average knowledge, and poor knowledge, respectively.
c) Attitude scale: The attitude of PHCWs will be measured by using the Likert scale. The scores are categorized into positive and negative attitude scores. Total 30 items with 5 Likert scores are included. The positive score of attitude scale for staff nurses, ANMs ranges from 91 to 150, and the negative score ranges from 30 to 90. For ASHA workers, the attitude scale contains ten items with five Likert scores. The positive score of the attitude scale for ASHA workers ranges from 31 to 50 and the negative score ranges from 10 to 30.
d) Skill checklist: Various skills will be assessed by using a skill checklist during the training programs like colostomy care, nasogastric tube insertion and tube feeding, urinary catheterization and catheter care, oral care, tracheostomy care, wound care, prevention of bedsores, subcutaneous injection procedures, lymphedema care. The checklist for skill assessment has yes/No questions. The research team will observe the participants using the checklist while performing the procedures to assess their skills.
e) Focus Group Discussion (FGD) among PHCWs from selected PHCs: questions will be asked regarding the facilitators and barriers in providing palliative care at the end of one year after implementing the palliative care training program. Thematic analysis will be used to evaluate the outcome of the focus group discussion.

### Statistical analysis

Descriptive statistics, inferential statistics, and thematical analysis will be used for summarising the data. Using descriptive and inferential analysis, demographic proforma, knowledge questionnaire, skill assessment questionnaire, case, and symptom identification checklist will be summarised by using SPSS 16.0. The Focus group discussion will be analyzed by using thematical analysis.

### Sample size calculation

**Sample size**

The expected sample size required for the study is given below:

$$n=\frac{Z^{2}\left(1-\frac{\alpha }{2}\right)\left(\mathrm{pq}\right)}{dp^{2}\;}=\frac{1.96\left(0.65\right)\;\times \;\left(1-0.65\right)}{{\left(0.05\times 0.65\right)}^{2}}=830$$

Where,

Z = 1.96 at = 5% level of significance,

p = proportion of Community Health workers who would implement = 65%

q = 1-p

d = precision at 5%

We propose to include all the PHCWs from the selected district of the southern part of India. As per the sample size calculation, the required sample size is 830, and presently 1440 PHCWs are there in the Udupi district. However, as we aim to cover the whole district PHCs and reach all needy populations, we will take 1440 participants for the capacity enhancement program, including new staff.

### Ethical considerations

Administrative permission will be obtained from Directorate General Health and Family Welfare, Karnataka state, to relieve ASHA workers and Community Health Workers for training on palliative care. Permission will be obtained from the District Health Officer and Medical Officer of all Primary Health Centres of Udupi District. Approval from Institutional Research Committee (IRC 239/2019) and Institutional Ethical Committee (IEC:164/2020) are obtained. The study is registered in the Clinical Trial Registry of India (CTRI) portal (CTRI/2020/04/024792). Informed written consent will be obtained from all the participants.

### Validity and reliability

The questionnaire will be tested for its validity and reliability by using split-half reliability. The module is prepared in English and translated into Kannada, which will be sent to five experts for validation. The questionnaires and module will be finalized after making necessary modifications as suggested by the validators.

### Limitations

The proposed capacity building of primary care workers follows the world health organizations’ recommendation of integration of palliative care into the public health system to achieve universal health coverage. Lack of cooperation from the primary health care workers may hinder the study. PHCWs may find difficulties in approaching the patients due to the varied distribution of cancer patients in a given geographical location. Lack of follow-up of cancer patients admitted and discharged from the different tertiary care hospitals other than research settings will also be the limitation of this study. Participants may face issues related to technology-enhanced learning, like the use of the mobile app.

### Dissemination of information

The study outcomes will be published through peer-reviewed journals. The results of this study will be communicated to the external funding body through a formal report.

### Study status

The study started in November 2020 and will continue until 2023. Capacity building of ASHA workers and ANMs will be initiated by April 2022.

## Conclusion

To the best of our knowledge, this will be the first study to assess the effectiveness of capacity building of primary care workers on the improvement of their symptom assessment and management in the Indian community setting. This research provides baseline information about palliative care needs in the community as well as possible facilitators and barriers for the implementation of the project. If this capacity enhancement is found to be effective, then the capacity-building program can be extended to state and national levels, so that palliative care services can be integrated at PHCs at affordable/free of cost.

This research could provide baseline information regarding palliative care needs in the community and even it could be a new community-based initiation taken at the doorstep of cancer patients to promote, restore, and maintain a person’s maximum level of comfort, function, and health, including care toward a dignified death.

## Data availability

### Underlying data

No data are associated with this article.

### Extended data

Figshare: Figshare: Demographic Performa, https://doi.org/10.6084/m9.figshare.19499072 ( Pai, 2022a).

Figshare: Participant information sheet and informed consent for ASHA worker, https://doi.org/10.6084/m9.figshare.19499075 ( Pai, 2022b).

Figshare: Participant information sheet and informed consent for staff nurses, https://doi.org/10.6084/m9.figshare.19499078 ( Pai, 2022c).

Data are available under the terms of the Creative Commons Attribution 4.0 International license (CC-BY 4.0).
