# Supplementary material for: Improving symptom assessment and management in the community through capacity building of primary palliative care: A study protocol of exploratory research
Source: F1000Res. 2022 Jul 1;11:733. [Version 1] doi: 10.12688/f1000research.111644.1 (PMC11391187; doi:10.12688/f1000research.111644.1)
Supplement: Peer review history

# Reviewer report: Reviewer response for version 1

**DOI:** 10.5256/f1000research.123364.r177647

**Published:** 2024-09-11

**Authors:** Neetha Kamath

**Affiliations:** 1 Department of Community Health Nursing, Nitte Usha Institute of Nursing Sciences, Nitte Deemed to be University, Mangalore, Karnataka, India

The literature review is extensive and need of the study is explained. The study addresses the current issues related to the problem. The methodology chosen meets the objective of the study. The ethics approval is obtained as per the ICMR guidelines. The sample size and data analysis plan is appropriate to the study protocol.

Is the study design appropriate for the research question?

Yes

Is the rationale for, and objectives of, the study clearly described?

Yes

Are sufficient details of the methods provided to allow replication by others?

Yes

Are the datasets clearly presented in a useable and accessible format?

Yes

Reviewer Expertise:

Home based care for menopausal women to address the issues of menopause

I confirm that I have read this submission and believe that I have an appropriate level of expertise to confirm that it is of an acceptable scientific standard.

## References

[1] : Developing Evidence-Based Clinical Guidelines in Palliative Care for Home Care Setting in India. *Indian J Palliat Care* .2020;26(3) : 10.4103/IJPC.IJPC_161_19 319-322 10.4103/IJPC.IJPC_161_19 33311873 PMC7725183

# Reviewer report: Reviewer response for version 1

**DOI:** 10.5256/f1000research.123364.r255915

**Published:** 2024-03-29

**Authors:** Sarah Mollman

**Affiliations:** 1 South Dakota State University, Brookings, South Dakota, USA

The aim and research objectives are clear. The background and significance (rationale) of the research is difficult to follow, skipping back and forth between points (example, rationale for the study population, use of mobile app, and importance of palliative care).

 The figures were very helpful in understanding your study and methods. I recommend including information about the security of the app. On page 7, it states it will be added to PHCW's mobile devices. Are these personal devices and what is the security if so? What is the security of the data in local and central storage as well as transferring between the two? How are you protecting the participants' privacy and confidentiality?

 Are the knowledge and attitude questionnaires new? If so, how were they developed? Did the process follow best practices? If not, include information on reliability and validity.

 Your population size is excellent and can lead to a strong study. How realistic is obtaining 58% of this population to participate in your study to reach the expected sample size? Is there literature to support 58% of a population completing a study? What is your goal for sample size of the focus groups?

Is the study design appropriate for the research question?

Yes

Is the rationale for, and objectives of, the study clearly described?

Partly

Are sufficient details of the methods provided to allow replication by others?

Partly

Are the datasets clearly presented in a useable and accessible format?

Not applicable

Reviewer Expertise:

Palliative care education, palliative care delivery to rural oncology patients.

I confirm that I have read this submission and believe that I have an appropriate level of expertise to confirm that it is of an acceptable scientific standard, however I have significant reservations, as outlined above.

# Reviewer report: Reviewer response for version 1

**DOI:** 10.5256/f1000research.123364.r146042

**Published:** 2022-08-15

**Authors:** Rakesh Garg

**Affiliations:** 1 Department of Onco-Anaesthesiology and Palliative Medicine, Dr BRAIRCH, All India Institute of Medical Sciences, Delhi, New Delhi, India

Improving symptom assessment and management in the community through capacity building of primary palliative care: A study protocol of exploratory research:

 The proposed study appears to have translational value in improving palliative care delivery and identifying gaps and role of grass root level workers who are already existing but need to be brought into the streamlining of palliative care.

 Please elaborate on various tools and instruments  - whether there are existing validated tools or new tools will be developed. In this case, elaborate on the process of tool development and its validation. Also emphasize on the compliance assurance of the ASAH cadres with regards to the implementation of various tools and apps.

Is the study design appropriate for the research question?

Yes

Is the rationale for, and objectives of, the study clearly described?

Yes

Are sufficient details of the methods provided to allow replication by others?

Partly

Are the datasets clearly presented in a useable and accessible format?

Yes

Reviewer Expertise:

Onco-Anaesthesiology and Palliative Medicine

I confirm that I have read this submission and believe that I have an appropriate level of expertise to confirm that it is of an acceptable scientific standard.
